# Comparison of vector elements and process conditions in transient and stable suspension HEK293 platforms using SARS-CoV-2 receptor binding domain as a model protein

**DOI:** 10.1186/s12896-023-00777-7

**Published:** 2023-03-07

**Authors:** Erica A. Green, Nathaniel K. Hamaker, Kelvin H. Lee

**Affiliations:** Department of Chemical and Biomolecular Engineering, University of Delaware, 590 Avenue 1743, Newark, Delaware, 19713 USA

**Keywords:** HEK293, SARS-CoV-2 receptor binding domain (RBD), EBNA1, Recombinant protein production, Process development, Viral vector production host

## Abstract

**Background:**

Mammalian cell lines are frequently used as protein expression hosts because of their ability to correctly fold and assemble complex proteins, produce them at high titers, and confer post-translational modifications (PTMs) critical to proper function. Increasing demand for proteins with human-like PTMs, particularly viral proteins and vectors, have made human embryonic kidney 293 (HEK293) cells an increasingly popular host. The need to engineer more productive HEK293 platforms and the ongoing nature of the severe acute respiratory syndrome coronavirus 2 (SARS-CoV-2) pandemic presented an opportunity to study strategies to improve viral protein expression in transient and stable HEK293 platforms.

**Results:**

Initial process development was done at 24 deep well plate (DWP) -scale to screen transient processes and stable clonal cell lines for recombinant SARS-CoV-2 receptor binding domain (rRBD) titer. Nine DNA vectors that drove rRBD production under different promoters and optionally contained Epstein-Barr virus (EBV) elements to promote episomal expression were screened for transient rRBD production at 37 °C or 32 °C. Use of the cytomegalovirus (CMV) promoter to drive expression at 32 °C led to the highest transient protein titers, but inclusion of episomal expression elements did not augment titer. In parallel, four clonal cell lines with titers higher than that of the selected stable pool were identified in a batch screen. Flask-scale transient transfection and stable fed-batch processes were then established that produced rRBD up to 100 mg/L and 140 mg/L, respectively. While a bio-layer interferometry (BLI) assay was crucial for efficiently screening DWP batch titers, an enzyme-linked immunosorbent assay (ELISA) was used to compare titers from the flask-scale batches due to varying matrix effects from different cell culture media compositions.

**Conclusion:**

Comparing yields from the flask-scale batches revealed that stable fed-batch cultures produced up to 2.1x more rRBD than transient processes. The stable cell lines developed in this work are the first reported clonal, HEK293-derived rRBD producers and have titers up to 140 mg/L. As stable production platforms are more economically favorable for long-term protein production at large scales, investigation of strategies to increase the efficiency of high-titer stable cell line generation in Expi293F or other HEK293 hosts is warranted.

**Supplementary Information:**

The online version contains supplementary material available at 10.1186/s12896-023-00777-7.

## Background

Mammalian cell lines are commonly used for viral vector and recombinant protein production at research and commercial scales. Cell lines such as Chinese hamster ovary (CHO), murine myeloma (NS0) and human embryonic kidney 293 (HEK293) are advantageous host platforms versus bacteria and yeast due to their ability to correctly assemble complex proteins, secrete them at high levels, and add human-like post-translational modifications (PTMs) [1–4]. Of these mammalian cell lines, HEK293 provides the additional benefit of conferring fully human PTMs that are less immunogenic [5–7]. Human-derived protein is crucial for initial characterization studies, and depending on the protein, may be required to ensure activity as a therapeutic [5, 8] or functionality in a medical device [9, 10]. Many therapies have been approved for commercial manufacturing in HEK293, including Nuwiq®, a recombinant factor VIII for hemophilia, and Luxturna®, an adeno-associated viral gene therapy for inherited retinal dystrophy [2].

Demand for HEK293-derived therapeutics is outpacing the rate of expression platform development as optimization of these systems is an ongoing, non-trivial task. Production processes can be transient, which require transfection of a DNA vector to initiate protein expression, or stable, in which a constitutive or inducible expression vector is integrated into the host genome [2, 11]. Careful design of DNA vectors is therefore a crucial step in developing highly productive cell cultures [12–15]. Once the best performing vector configuration is selected, process conditions can also be optimized to increase titer [2, 16].

Vector elements can be optimized during transient platform development, and they influence protein expression by two main mechanisms: direct regulation of the expression machinery for the gene of interest or indirect regulation by manipulation of the cellular environment [16, 17]. When evaluating elements that directly regulate the transgene, screening promoters is important, as their performance can be host cell and target protein dependent. The use of strong promoters, such as the human cytomegalovirus immediate early (CMV-IE) and CAG promoters, leads to the highest protein expression in many contexts [18–20], but in some cases, a weaker promoter may be required for optimal gene control [16]. Once a promoter is chosen, expression may be further augmented by modifying culture conditions such as process temperature [10, 20–22], cell density [23], and transfection parameters [8, 23, 24]. A strategy for making the cellular environment more favorable to recombinant protein production is expression of viral elements or chromatin regulating domains that promote episomal maintenance of DNA vectors post-transfection [25, 26]. One application of this approach uses Epstein-Barr virus (EBV) nuclear antigen 1 (EBNA1) to activate episomal replication and enable persistence of vectors containing the EBV origin of plasmid replication (oriP) [11, 27–30]. Expression of full-length EBNA1 or a repeat-less version of EBNA1 lacking most of its Gly-Gly-Ala repeat region functions by tethering plasmid DNA to the host cell chromosomes via oriP during cell division and supports plasmid replication in certain cell types [31, 32].

Optimization of vector design and screening of process parameters is also performed during stable platform development. The strength of the promoter driving the gene of interest and the ability of the expression cassette to sustain long-term transcriptional activity once integrated into the host cell genome are crucial for stable expression [16, 17]. Non-coding regulatory elements such as matrix attachment regions (MARs) can increase chromatin accessibility and insulate adjacent sequences from positional effects, which generally confers improved expression when placed upstream and/or downstream of the transgene [19, 33, 34]. After stable cell lines are established, fed-batch process parameters such as feed schedules and specific nutrient set points can be tested for further optimization [35, 36].

Studies described in this work focus on systematically evaluating contributions from both vector design and process parameters to maximize productivity for transient and stable HEK293 platforms. Like others, we use the severe acute respiratory syndrome coronavirus 2 (SARS-CoV-2) receptor binding domain (RBD) as a model protein [10]. Here, nine vectors transiently expressed recombinant RBD (rRBD) from the human CMV-IE (CMV), CAG, or simian virus 40 early (SV40) promoter with and without co-expression of EBNA1 variants were screened for productivity in Expi293F cells cultured at 37 °C or 32 °C. In parallel, a CAG-driven, rRBD cassette flanked by two MAR elements was used to develop clonal cell lines for protein production in fed-batch format. We observed that CMV-driven rRBD expression at 32 °C led to the highest transient protein titer. Surprisingly, co-expression of EBNA1 did not augment transient rRBD expression in any vector context. Stable fed-batch titers were significantly higher than those of the highest yielding transient system, and the developed process provides a baseline that can be further optimized for large-scale protein or viral vector production.

## Methods

### Cell line and culture conditions

Expi293F cells (ThermoFisher A14527) were maintained in an Infors Multitron HT (25 mm throw) kept at 37 °C, 80% relative humidity (RH), and 8% CO_2_. Cells were cultured in the native Expi293 medium (ThermoFisher) for transient protein expression, and BalanCD HEK293 medium (Irvine Scientific) supplemented with 4 mM GlutaMAX (ThermoFisher) for stable cell line development and fed-batch production. Cultures for routine cell maintenance were seeded at 0.3–0.4 × 10^6^ cells/mL in non-baffled 125 mL shake flasks (Fisher Scientific) at a 20–30 mL working volume, shaken at 120 rpm, and passaged every 3 days. Well plate (WP), deep well plate (DWP), and bioreactor tube cultures for protein production or clonal cell line scale-up were shaken at 200 rpm, and production shake flask cultures were shaken at 120 rpm. Viable cell density (VCD) and cell viability were monitored using trypan blue exclusion with a Vi-Cell XR (Beckman Coulter). Cells were grown for ≥ 3 passages post-thaw prior to protein production or cell line development.

### Plasmid construction

Plasmids (DNA vectors) were constructed by Gibson assembly or restriction digestion and insertion from combinations of gBlock synthetic DNA fragments (Integrated DNA Technologies), restriction enzyme digestion products, and polymerase chain reaction (PCR) products. PCRs were performed using Phusion Hot Start Flex 2x Master Mix or Phusion High-Fidelity PCR Master Mix with HF buffer (New England Biolabs) following the manufacturer’s protocol. Restriction enzymes and buffers for digests were purchased from New England Biolabs and used per the manufacturer’s instructions. Digestion and PCR products were cleaned up with the QIAquick PCR Purification Kit (Qiagen) prior to plasmid assembly. Gibson assemblies were performed using NEBuilder HiFi DNA Assembly Master Mix (New England Biolabs) and ligations were performed using T4 DNA ligase (Invitrogen) following the respective manufacturer’s protocol. Plasmids were propagated in One Shot TOP10 Chemically Competent *E. coli* (ThermoFisher) and isolated using an EndoFree® Plasmid Maxi, Plasmid Plus Midiprep, or QIAprep Spin Miniprep kit (Qiagen) per the respective manufacturer’s protocols for sequence verification and use in transient production or stable cell line development.

**Fig. 1:**
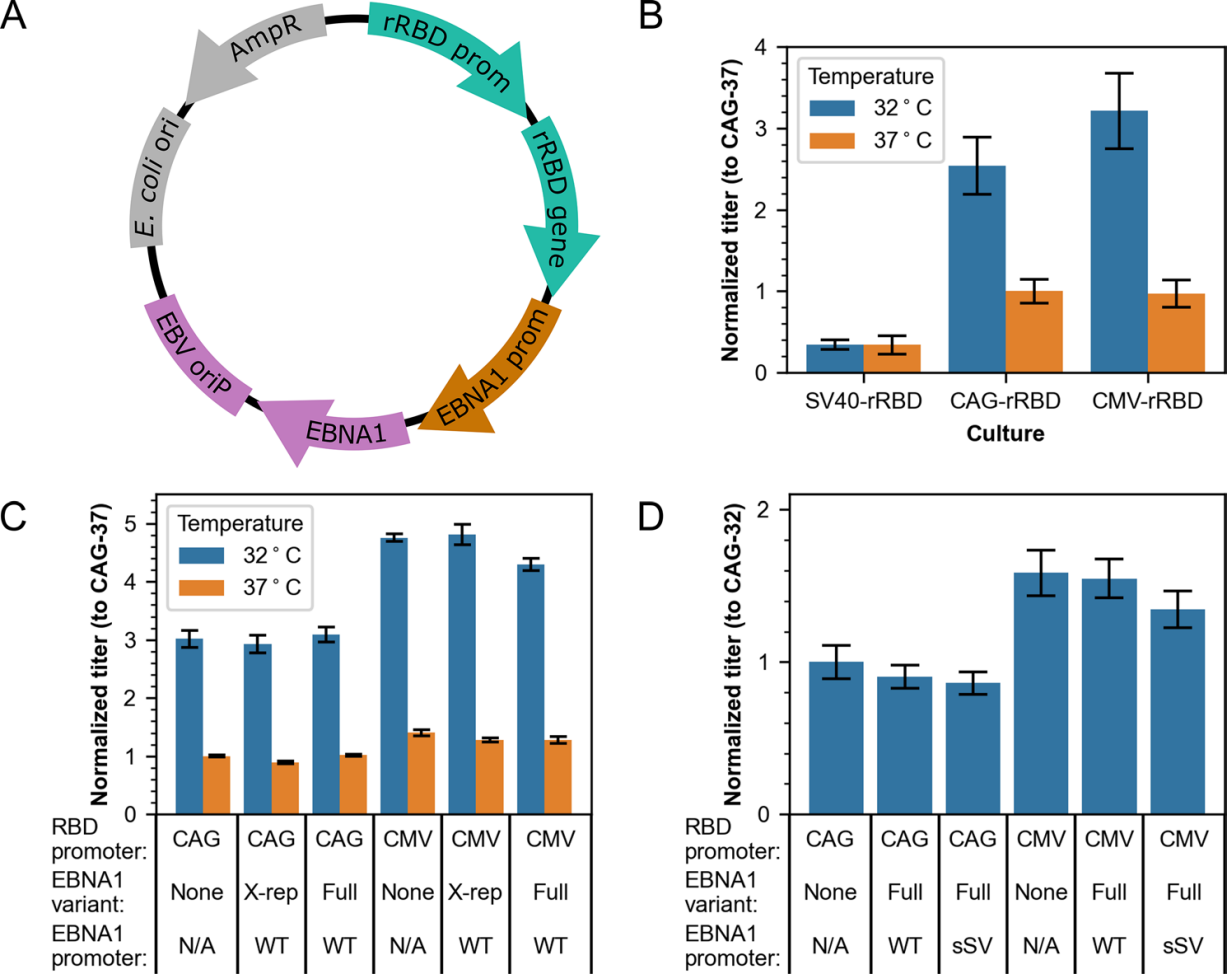
Transient plasmid screen at deep well plate-scale to optimize rRBD titer. **(A)** Schematic summarizing the elements on the transient expression vectors. The histidine-tagged rRBD gene and associated promoter (rRBD prom) are teal, EBV episomal elements are purple, the optional additional promoter (EBNA1 prom) for EBNA1 is brown, and the ampicillin resistance gene (AmpR) and origin of replication for *Escherichia coli* (*E. coli* ori) are grey. **(B)** Normalized BLI titers for the study comparing culture temperatures (32 °C vs. 37 °C) and promoters (SV40, CAG, CMV) for rRBD expression. **(C)** Normalized BLI titers for the study comparing culture temperatures (32 °C vs. 37 °C) and EBNA1 gene variants (no EBNA1 [None], repeat-less EBNA1 [X-rep], and wild-type EBNA1 [Full]). **(D)** Normalized BLI titers for the study comparing EBNA1 expression cassette variants (no EBNA1 [N/A], wild-type EBNA1 [WT] and synthetic SV40 driven wild-type EBNA1 [sSV]). Error bars show SEM for biological and technical duplicates. Corresponding absolute titers used for normalization are shown in Table S2

The rRBD expression cassette contained an N-terminal human serum albumin secretion signal, residues 319–599 of the SARS-CoV-2 spike protein, and a C-terminal 8X histidine tag. Plasmids used for transient rRBD protein production were constructed in series. First, the CMV-rRBD plasmid was constructed by Gibson assembly. The CAG-rRBD and SV40-rRBD plasmids were then built by digestion and ligation reactions in which the CMV promoter was replaced with the CAG or SV40 promoter. Parts of the EBNA1 and oriP sequences were amplified by PCR from the genome of EBV (VR-1492; American Type Culture Collection) and inserted into pCR®-Blunt II-TOPO vectors (Invitrogen) per the manufacturer’s protocol. Restriction digestion and insertion was then used to insert oriP/wild-type EBNA1 into the CMV-rRBD plasmid and oriP/repeat-less EBNA1 into the CAG-rRBD and CMV-rRBD plasmids. The CAG-rRBD plasmid containing the oriP/wild-type EBNA1 was derived from pCXLE-EGFP (Addgene plasmid # 27082). Insertion of a synthetic SV40-derived (sSV40) promoter driving EBNA1 expression was performed by Gibson assembly following digestion of the CAG-rRBD-wtEBNA1 and CMV-rRBD-wtEBNA1 plasmids. A schematic depicting the design of the transient plasmids is shown in Fig. 1A.

**Fig. 2:**
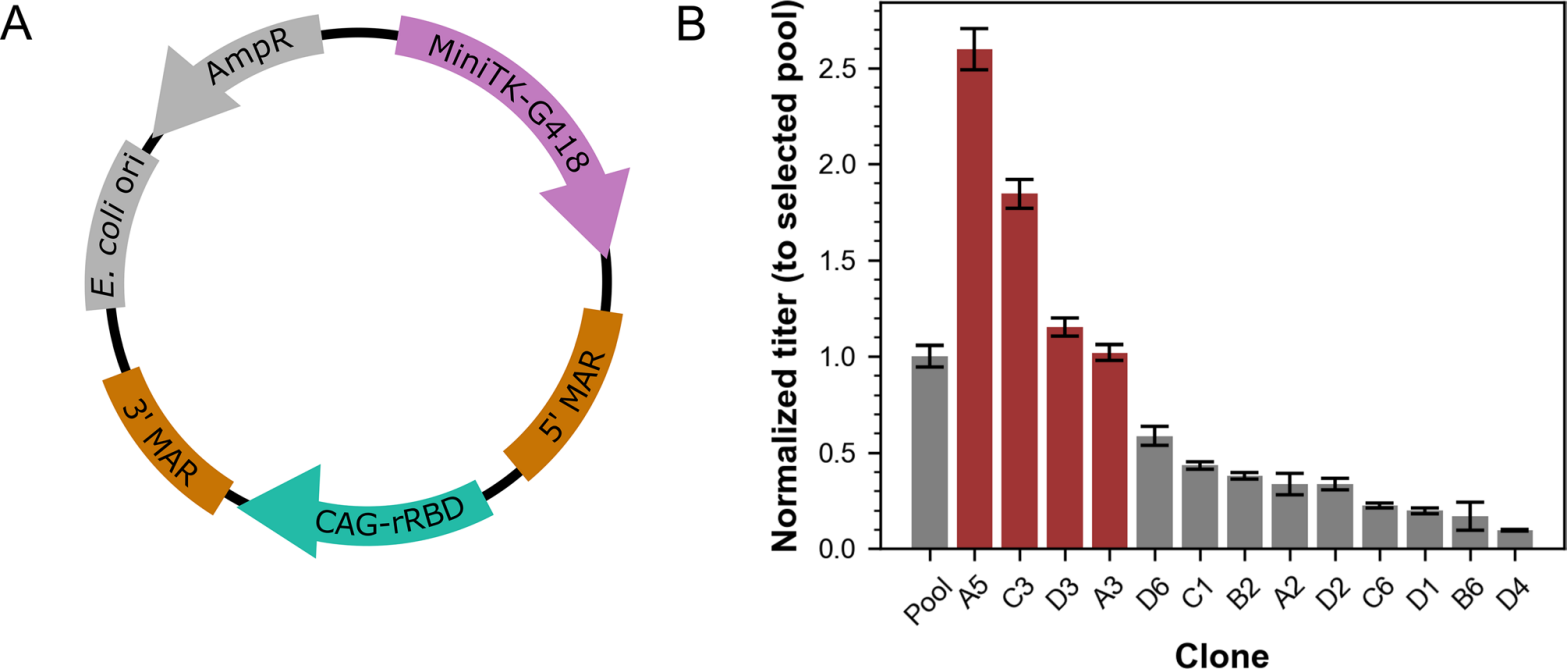
rRBD-producing stable clone generation and batch screen. **(A)** Schematic of the stable expression plasmid. The Geneticin (G418) resistance gene driven by the minimal herpes simplex virus thymidine kinase promoter (MiniTK) is purple, the rRBD gene driven by the CAG promoter is teal, the insulating matrix attachment region (MAR) elements are brown, and the ampicillin resistance gene (AmpR) and origin of replication for *Escherichia coli* (*E. coli* ori) are grey. **(B)** rRBD BLI titer values were normalized relative to the selected pool. Bars for clones with normalized titer > 1 that were chosen for fed-batch process development and scale-up are red, and bars for the pool or clones with normalized titer < 1 are grey. Error bars show SEM for technical replicates of singlet cultures. The corresponding absolute titer used for normalization is shown in Table S2

The plasmid used for rRBD stable cell line development (pNeo-CAG-rRBD) was built using Gibson assembly and contained the same rRBD expression sequence as the transient plasmids. Once assembled, the plasmid contained the rRBD expression cassette flanked by the human β-interferon MAR [33, 34] on the 5’ end and the potato ST-LS1 MAR [37, 38] on the 3’ end, and a Geneticin (G418) resistance cassette. A schematic depicting the design of the stable plasmid is shown in Fig. 2A.

Restriction digestion between the PstI and NsiI sites was used to remove EBNA1 from pCXLE-EGFP (Addgene #27082) and build pCX-EGFP, both of which express enhanced green fluorescent protein (eGFP).

### Transient transfection for rRBD production

Expi293F cells maintained in shake flasks as described above were seeded at 0.3–0.4 × 10^6^ cells/mL in Expi293 expression medium and cultured in 125 mL, 500 mL, or 1 L flasks (Fisher Scientific or Corning) in working volumes of 30 mL, 100 mL, and 200 mL, respectively. On passage day 3 (the day preceding transfection), cells were re-seeded at 2.5–3.0 × 10^6^ cells/mL in fresh Expi293 medium. On transfection day, cells were re-suspended at 3.0 × 10^6^ cells/mL in fresh Expi293 medium. Cells were cultured in 24 DWPs (Corning) at 2.5 mL working volume and covered using Breathe-Easy sealing membranes (Sigma-Aldrich) or cultured in 125 mL flasks (Fisher Scientific) at 25 mL working volume.

Complexation of plasmid DNA with Expifectamine 293 reagent (ThermoFisher) was performed in Opti-Plex complexation buffer (ThermoFisher) following the manufacturer’s protocol. In brief: 1 µg of plasmid DNA per mL of culture and 3.2 µL of Expifectamine per mL of culture were added to separate aliquots of Opti-Plex buffer, and then combined for a 15 min complexation prior to addition to cells. Following transfection, cells were cultured at 37 °C for 30 min, and then cultured at production temperature (32 or 37 °C, depending on the batch).

Expifectamine 293 enhancers 1 and 2 were added to the cultures 22 h post-transfection per the manufacturer’s instructions. Transient protein production was terminated when cell viability was < 80% as measured on the Vi-Cell XR. To harvest the DWP and flask culture supernatants for titer analysis, the cultures were spun at 1,000 g for 5 min to remove cells. Flask supernatants underwent an additional spin at 15,000 g for 20 min to remove debris and clarified using 0.22 μm syringe filters (MilliporeSigma). All samples were stored at -20 °C.

### Generation of rRBD stable pool and clonal cell lines

Expi293F cells were directly adapted to BalanCD HEK293 medium and maintained in 125 mL shake flasks as described above. After eight passages in BalanCD HEK293 medium, 6 × 10^6^ cells were transfected with 10 µg of pNeo-CAG-rRBD stable expression vector linearized with PvuI-HF (NEB). Briefly, cells and DNA were resuspended in Solution SF and Supplement 1 (Lonza) to a total volume of 100 µL and transfected using an Amaxa 4D-Nucleofector (Lonza) with program FS-100. Cells were diluted in 0.5 mL warm BalanCD HEK293 medium, transferred to a non-treated 6-well plate (Corning) to recover for 40 min without agitation, and then resuspended in 20 mL BalanCD HEK293 medium for culture in a 125 mL shake flask.

On day 2 post-transfection, cells were selected with 400 µg/mL Geneticin (G418; ThermoFisher). Once the VCD was > 1 × 10^6^ cells/mL, cells were selected with 800 µg/mL G418, and selection pressure was maintained until the culture displayed VCD and viability similar to non-transfected host cells. Throughout selection, cells were passaged to 0.3 × 10^6^ cells/mL if VCD was > 1 × 10^6^ cells/mL, or underwent a full media exchange every 3 days, depending on cell density. After recovery from selection, cells from the selected pool were plated on semi-solid ClonaCell TCS medium (StemCell Technologies) supplemented with 5% tryptose phosphate broth (Millipore Sigma). Three dilutions of cells (50, 25, and 12.5 cells/mL) in semi-solid medium were prepared and dispensed into duplicate wells of a 6-well plate (Corning) for static incubation at 37 °C.

### Expansion of rRBD clones and batch culture

Colonies were picked on day 10 post-plating following verification of clonality by inspection on an inverted microscope (Evos™ XL). Clonal cell lines were cultured in BalanCD HEK293 medium supplemented with Accumax™ (MilliporeSigma) at 100x dilution in non-tissue treated 96 WPs, non-tissue treated 24 WPs, and 24 DWPs (Corning) covered with a Breathe-Easy sealing membrane for initial outgrowth. Specifically, cells were maintained in a 96 WP (200 µL working volume) for 6 days post-picking. Any colonies that had grown per inspection on an inverted microscope were transferred to a 24 WP (700 µL working volume) for 3 days, and then a 24 DWP (2 mL working volume) for two additional 3-day passages.

Subsequently, any clones with acceptable growth phenotypes (VCD > 1.0 × 10^6^ cells/mL) were seeded for a batch production screen. Cells were seeded at 0.3 × 10^6^ cells/mL in 24 DWP format (2 mL working volume) and cultured for 7 days at 37 °C. Supernatant samples were saved at -20 °C for titer analysis after centrifugation to remove cells.

### Fed-batch culture of rRBD clones

Clonal rRBD producing cell lines were seeded at 0.3 × 10^6^ cells/mL in 125 mL shake flasks at a 30 mL working volume and cultured at 37 °C. Samples were taken daily to measure VCD and cell viability with a Vi-Cell XR, and glucose and lactate levels with a YSI 2950 biochemistry analyzer (Xylem). Starting on day 3, cells were fed 3.3% v/v BalanCD HEK293 feed (Irvine Scientific) supplemented with 4 mM GlutaMAX and bolus additions of 45% glucose solution (Sigma-Aldrich) to reach 7 g/L of glucose. Batches were terminated once cell viability was < 80%. Supernatants were harvested for titer analysis using the same protocol as for transient batch harvest.

### Post-harvest quantification of rRBD titer by bio-layer interferometry (BLI)

The Octet RED96e system (Sartorius) was used for rRBD protein quantification in all supernatant samples using Anti-Penta-HIS (HIS1K) biosensors (Sartorius #18-5120). Supernatant samples were diluted 2- or 5-fold in phosphate buffered saline (PBS; Fisher Scientific) for analysis. HIS1K biosensors were hydrated for ≥ 10 min in a blend of production medium (Expi293 or BalanCD HEK293) and PBS to match the supernatant:PBS ratio in the sample matrix. During analysis, the sample plate was maintained at 30 °C and 1000 rpm. Sample concentrations were interpolated from an unweighted 4-parameter logistic (4PL) regression model for serial dilutions of purified rRBD internal standard material.

The internal standard was produced by transient transfection of the CAG-rRBD-wtEBNA1 plasmid into Expi293F cells as described above, purified using the Ni-NTA Fast Start Kit (Qiagen), and then buffer exchanged into PBS using Amicon Ultra-15 Centrifugal Filter Units (MilliporeSigma) with a 10 kilodalton (kDa) cutoff. Standard concentration was measured by A280 absorbance on a DS-11 FX+ (DeNovix) using a 32.6 Da molecular weight (un-glycosylated sequence estimate) and 33,350 M^− 1^ cm^− 1^ extinction coefficient.

### Post-harvest quantification of rRBD titer by enzyme-linked immunosorbent assay (ELISA)

A Human SARS-CoV-2 RBD ELISA Kit (ThermoFisher) was used for quantification of rRBD in all transient and fed-batch samples produced at flask-scale. Samples were diluted 1,000,000-fold to fit within the standard curve based on BLI results. Assay steps were executed following the manufacturer’s protocol, and after stopping the colorimetric reaction, absorbance values were measured using a SpectraMax i3x plate reader (Molecular Devices). Sample concentrations were interpolated from a 4PL regression model for serial dilutions of the rRBD standard provided with the ELISA kit.

### rRBD purification, digestion, and sodium dodecyl-sulfate polyacrylamide gel electrophoresis (SDS-PAGE) analysis

Clarified rRBD supernatant samples from the transient and stable fed-batch cultures were purified for gel analysis using a Ni-NTA Spin Kit (Qiagen) under native conditions. Spin columns were equilibrated, loaded with 3 × 600 µL harvested supernatant, washed, and eluted per the manufacturer’s protocol. Samples were buffer exchanged into PBS using 10 kDa Amicon Ultra-0.5 Centrifugal Filter Units (MilliporeSigma). rRBD concentrations were measured by A280 absorbance.

Purified rRBD samples were buffer exchanged into molecular biology grade water (Fisher Scientific) using 10 kDa Amicon Ultra-0.5 Centrifugal Filter Units (MilliporeSigma). rRBD concentrations were measured by A280 absorbance. 15 µg of rRBD was then denatured and digested with PNGaseF (Promega) based on the manufacturer’s protocol. In brief, rRBD was denatured at 95 °C for 10 min in the presence of dithiothreitol (DTT; New England Biolabs) and sodium dodecyl-sulfate (SDS; Bio-Rad). PNGase F digestion with 1U/µL enzyme was performed in the presence of sodium phosphate pH 7.4 (Fisher Scientific) and Triton X-100 (MilliporeSigma) at 37 °C for 4 h.

Purified rRBD samples diluted with 2x Laemmli sample buffer (Bio-Rad) supplemented with 120 mM DTT (New England Biolabs) were boiled for 5–10 min. 1 µg of intact protein or PNGaseF digested protein was loaded into a 4–15% Mini-PROTEAN® TGX™ gel (Bio-Rad). 5 µL of Precision Plus Protein™ Unstained Protein Standards (Bio-Rad) was used as a molecular weight marker. The gel was run for 50 min at 150 V and then stained with SYPRO® Ruby (ThermoFisher). Fix and wash buffers were prepared per the manufacturer’s protocol, and a modified rapid protocol was executed for gel fixing, staining, and washing while rocking the gel on an orbital shaker. In brief, the gel was shaken in fresh fixing buffer 2 × 15 min, stained for 3 h, and shaken in fresh wash buffer 2 × 15 min. Gels were imaged on a Typhoon FLA 7000 (Cytiva) using the 473 nm laser and 580 nm emission filter.

### Droplet digital PCR (ddPCR) analysis for transgene copy number in clonal cell lines

Genomic DNA (gDNA) was extracted from 5 × 10^6^ cells of each rRBD-expressing clone using a QIAmp DNA Mini Kit (Qiagen) per the manufacturer’s protocol. Samples were digested prior to ddPCR with 1 unit enzyme per 20 µL of digest using HindIII in rCutSmart buffer at 37 °C (rRBD set 2/GAPDH samples) or BstYI in r2.1 buffer at 60 °C (rRBD set 1/RPP30 samples) for 2 h (all reagents from New England Biolabs). Multiplexed ddPCR was performed on a QX ONE system (Bio-Rad) using 2x ddPCR Supermix for Probes without dUTP (Bio-Rad). Reactions were prepared with 900 nM primer, 250 nM probes, and 36–48 ng gDNA and run using the default copy number variation (CNV) protocol. rRBD copies per diploid genome were calculated after assaying with rRBD set 1/RPP30 or rRBD set 2/GADPH (Table S1). Copy numbers used for *GAPDH* and *RPP30* as reference genes were 3 and 2, respectively.

### eGFP expression in batch and long-term culture format and monitoring of expression by flow cytometry

Expi293F cells were transfected with pCXLE-EGFP or pCX-EGFP with Expifectamine using an identical protocol to that used for rRBD transient expression. Transfected cells were cultured at 32 or 37 °C in bioreactor tubes (MilliporeSigma) at a 7.5 mL working volume. On day 2 post-transfection, cells from the 37 °C cultures were used to seed 24 DWP cultures at 0.4 × 10^6^ cells/mL for monitoring of eGFP expression levels over extended culture. Samples for flow cytometry were taken daily from batch and extended cultures. Batch cultures were terminated on day 6, and extended cultures were passaged to 0.4 × 10^6^ cells/mL every 3 days until termination on day 30.

Flow cytometry data was acquired on an Accuri C6 Plus flow cytometer (BD) equipped for detection of eGFP (488 nm laser, 533/30 emission). Gates were set using untransfected Expi293F host cells (Figure S1). All data analysis was performed using BD CSampler™ Plus Software v1.0.27.1.

### Statistical analysis

Data were processed and figures were generated using Python (v 3.10). Data in bar graphs or line plots are presented as mean ± the standard error of the mean (SEM) propagated through technical replicates, biological replicates, and any mathematical operations (e.g., normalization) as appropriate for each data set. Statistical significance of pairwise comparisons in each data set was calculated by Tukey’s post-hoc test. A p-value of < 0.05 was considered significant.

## Results

### Transient rRBD vector screen #1: culture temperatures and rRBD promoters

Initial evaluation of vectors for transient production focused on varying the culture temperature post-transfection and the promoter driving rRBD expression. Cultures were harvested when viability was < 80%, which occurred 6 days post-transfection for cultures grown at 32 °C and 3 days post-transfection for cultures grown at 37 °C (Figure S2). Normalized titers of harvest day supernatant samples relative to CAG-driven rRBD expression at 37 °C are shown in Fig. 1B. At 37 °C, transfection of vectors with CAG and CMV-driven rRBD expression led to similar titers (p > 0.05, Tukey test), while SV40-driven expression resulted in 2-fold lower rRBD yield. Titers from SV40 promoter driven expression were not different at 32 °C vs. 37 °C (p > 0.05, Tukey test). However, titers from the cultures transfected with the CAG and CMV-driven rRBD expression vectors were higher at 32 °C vs. 37 °C, with CMV-driven expression increasing over 3-fold on average for cultures grown at the lower temperature.

### Transient rRBD vector screen #2: EBNA1 gene variants

Subsequent experimentation focused on further study of the temperature-dependent titer increase observed for CAG and CMV constructs and whether rRBD expression could be augmented by co-expression of EBV elements in *cis*. The CAG-rRBD and CMV-rRBD vector versions tested either did not contain EBV elements or contained oriP with a variant of the wild-type EBNA1 gene (full-length or repeat-less), for a total of six vectors. Cultures grown at 37 °C and 32 °C were harvested on day 3 and day 6 post-transfection, respectively. Titers normalized to CAG-driven rRBD expression at 37 °C for harvest day samples are shown in Fig. 1C. As in the previous study, lowering the rRBD production temperature to 32 °C led to promoter-dependent titer increases, with a greater titer increase observed for CMV-rRBD vectors (~ 4.5-fold) than for CAG-rRBD vectors (~ 3-fold). At 37 °C, titers from CMV-driven expression were again not significantly different than those from CAG-driven expression (p > 0.05, Tukey test). Most notably, expression of rRBD from episomal vectors containing the full-length or repeat-less EBNA1 gene did not lead to significantly higher titers versus expression from vectors that did not contain EBNA1, regardless of the promoter driving rRBD expression or culture temperature (p > 0.05, Tukey test, for all four rRBD promoter and culture temperature groupings).

### Transient rRBD vector screen #3: EBNA1 expression cassettes

An additional transient vector screen was performed to further probe the relationship between transient expression of rRBD and EBNA1 in *cis*, with a focus on whether placing additional promoter sequences in front of EBNA1 would enable an increase in rRBD titer [31]. CAG-rRBD and CMV-rRBD vector derivatives with and without the sSV40 promoter placed upstream of the full-length, wild-type EBNA1 gene were tested alongside those without EBV elements, for a total of six vectors. Cultures were grown at 32 °C and harvested 6 days post-transfection. Normalized titers relative to CAG-driven rRBD expression at 32 °C are shown in Fig. 1D. As in previous DWP batches, all CMV-rRBD vectors drove higher rRBD expression at 32 °C than all CAG-rRBD vectors (p < 0.05, Tukey test). On average, titers were lowest from cultures transfected with vectors containing the sSV40-EBNA1 cassette versus the WT EBNA1 or no EBNA1 cassette.

### Transient eGFP expression with and without EBNA1 co-expression over short- and long-term culture

The EBNA1 expressing cultures had either similar or lower eGFP + populations and mean fluorescence intensities (MFIs) during 6-day batches when cells were cultured at either 37 °C or 32 °C (Figure S3A/B). Interestingly, cultures with in *cis* EBNA1 co-expression maintained eGFP expression levels above the ~ 0.5% eGFP + stable baseline for longer than eGFP only expressing cells during extended culture (Figure S3C/D).

### Selection of rRBD stable pool, establishment of clonal cell lines, and batch titer screen

The rRBD expressing pool fully recovered from G418 selection 17 days post-transfection (Figure S4). Cells subsequently diluted for single-cell cloning expanded into colonies large enough to be picked after 10 days of incubation. Of the 72 clones picked for expansion, 13 had acceptable growth phenotypes after initial scale-up to 24 DWPs.

Titers from the 7-day batch screen of 13 rRBD clonal cell lines were normalized against that of the selected pool (Fig. 2B). Four clones, A5, C3, D3, and A3 had normalized titers greater than 1 and were chosen for evaluation in fed-batch culture format.

### Transient process scale-up to shake flasks

The EBNA1-free and wild-type EBNA1 containing rRBD-CAG and rRBD-CMV vectors were transfected into cultures grown in shake flasks to confirm that trends seen in the DWP screen held at larger scales. Transfection parameters used in the 2.5 mL DWP batches were scaled up proportionally for transfection of a 25 mL culture and subsequent production at 32 °C. VCD and viability curves from all four conditions trended such that peak VCD was reached on day 3 or 4, and viability declined below 80% by day 6 (Figure S5). Co-expression of EBV elements did not have an adverse effect on cell growth profiles.

**Fig. 3:**
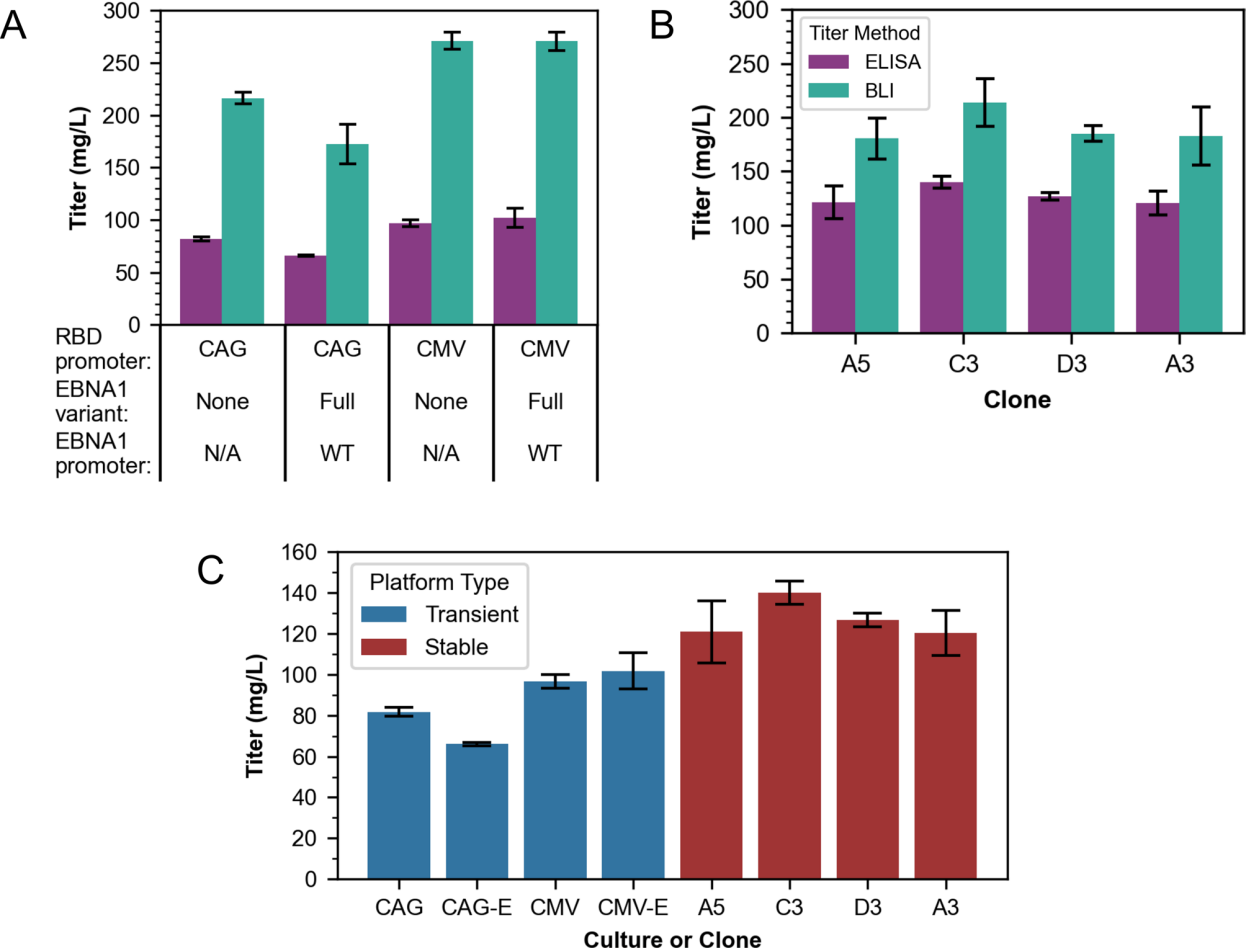
Titers for flask-scale batches measured by orthogonal analytical methods. Absolute titers **(A-** transient, **B-** stable**)** measured on harvest day supernatant samples by ELISA and BLI for the flask-scale batches. **(C)** A comparison of absolute titers measured by ELISA for all flask batches per transient culture (-E denotes plasmids with wild-type EBNA1) or stable clone is also shown. Error bars show SEM for transient biological duplicates and fed-batch biological triplicates with error propagated for technical replicates (n = 2 for BLI, n = 3 for ELISA)

Trends for the flask-scale batch titers by ELISA and BLI led to conclusions consistent with those from the DWP-scale BLI titers (Fig. 3A). Expression of rRBD under the CMV promoter led to ≥ 1.2-fold higher final yield than under the CAG promoter at 32 °C, and co-expression of EBV elements did not increase rRBD titers. Titers from expression of CAG-rRBD constructs decreased when EBV elements were co-expressed (p < 0.05, Tukey test), and expression of CMV-rRBD constructs with and without EBV elements did not lead to significantly different final protein titers (p > 0.05, Tukey test).

### Fed-batch culture of top clones at 125 mL flask-scale

Clones A5, C3, D3, and A3 were cultured in fed-batch format at flask-scale to establish a baseline for the performance of antibiotic-selected rRBD producer cell lines derived from Expi293F (Figure S6). During the first 6 days of culture, all clonal cell lines maintained high viability (> 96%), total glucose consumption rates increased, and lactate levels increased as the cells grew exponentially. As the cultures approached and entered stationary phase, viabilities dropped to 90–95%, glucose consumption rates steadied, and lactate levels decreased. The trajectory of lactate level increases and VCD/cell viability decreases leading up to batch termination were similar for all cultures, but day-to-day trends were clone dependent. Batches were terminated on day 21 for all replicates of A3 and C3, and day 24 for all replicates of D3. Less uniformity was observed in the culture behavior of clone A5, as replicates 1 and 2 reached < 80% viability on day 20, while replicate 3 did so on day 21.

The ranking of clones by mean fed-batch titer from highest to lowest (C3, D3, A3, A5; Fig. 3B) was different than by clone screen batch titer (A5, C3, D3, A3; Fig. 2B). Copy number analysis indicated that the clones had ~ 3–6 copies of the rRBD gene stably integrated (Figure S7), but the ranking of clones by most to least copies (C3, A5, D3, A3) did not exactly match the mean titer rankings for the fed-batch or 24 DWP screen. Based on these data, C3 was the top-performing stable clone in fed-batch format (140 mg/L) by ELISA and had the highest rRBD copy number.

### Orthogonal titer method comparison for flask-scale batches

rRBD titers for flask-scale transient and stable batches were measured by ELIS in addition to BLI. These orthogonal analyses were performed because we observed that the BLI measurements were sensitive to dilution (Figure S8) and media-dependent matrix effects (Fig. 3A/B). Despite the discrepancy in absolute titers between assays, normalized titer values were similar per transient process relative to CAG-rRBD at 32 °C and per individual clone relative to A3 (p > 0.05, Tukey test, Figure S9A/B). Taken together, these observations show that relative titer trends hold between assays for each process type, but matrix effects must be evaluated to determine whether samples in different cell culture media compositions can be compared using a given assay. Inter-process titer comparisons in this work can be drawn from the ELISA measurements taken on more dilute samples (Fig. 3C), that show that the most productive stable fed-batches produced 1.4-fold more rRBD than was produced by the highest yielding transient batches.

### Gel analysis of transiently- and stably-produced rRBD for quality analysis

**Fig. 4:**
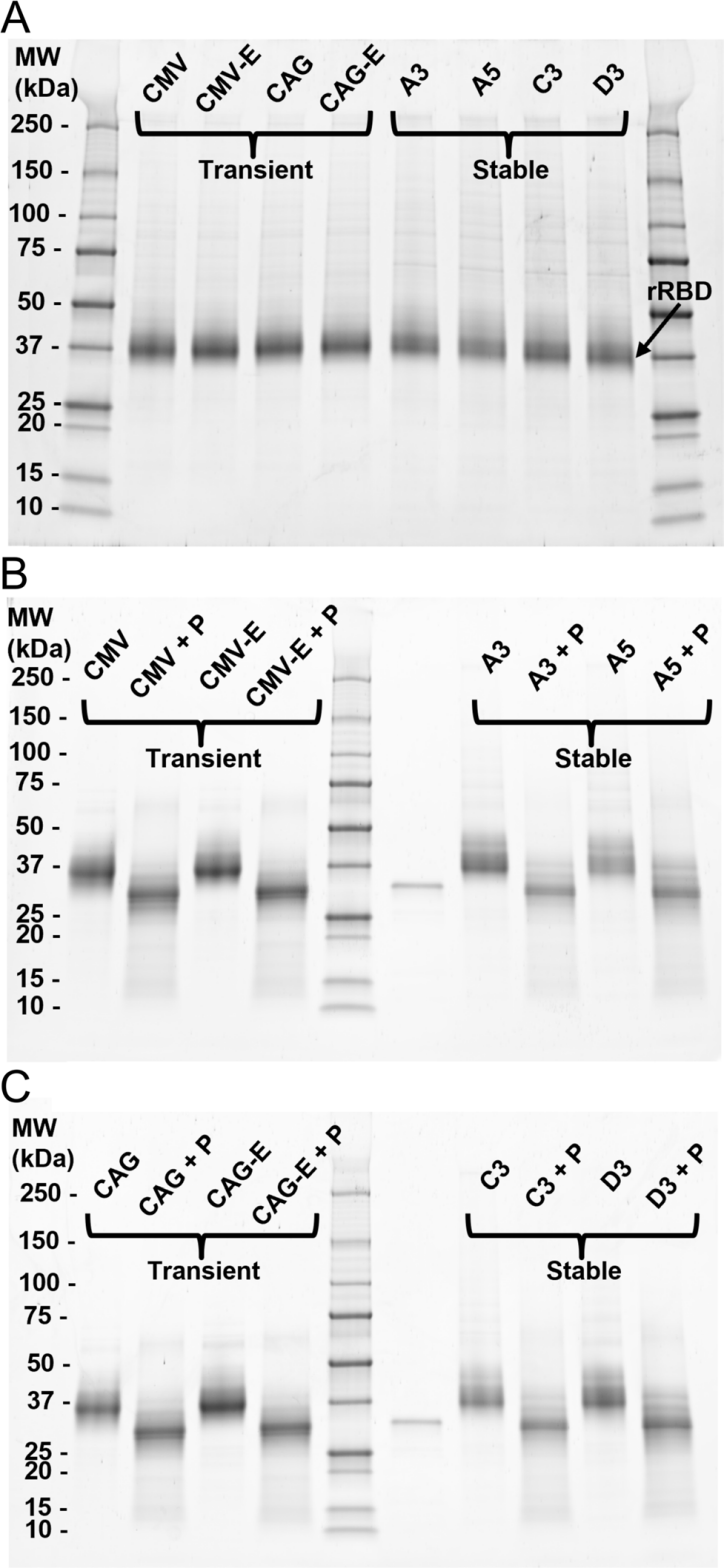
Sodium dodecyl sulfate–polyacrylamide gel electrophoresis (SDS-PAGE) quality analyses of purified rRBD produced in flasks. **(A)** SDS-PAGE analysis of purified rRBD samples transiently expressed or stably expressed. Transiently produced rRBD is marked by the promoter driving rRBD expression (CAG or CMV) and whether the oriP/wild-type EBNA1 are present (CAG-E or CMV-E). Stably produced rRBD is marked by clone. **(B/C)** SDS-PAGE analysis of purified rRBD samples (marked by transient culture or stable clone) without and with PNGaseF digestion (+ P). Samples were run across two gels with transiently produced samples on the left, a molecular weight ladder and PNGase F control in the middle, and stably produced samples on the right

SDS-PAGE was performed to determine qualitatively if any differences were evident between rRBD material produced from transient and stable batches (Fig. 4). Reduced protein material from both processes was ~ 36 kDa, which was greater than the theoretical molecular weight (32.6 kDa) predicted by the amino acid sequence alone (Fig. 4A). Analysis of PNGase F digested rRBD samples alongside intact protein showed that the molecular weight of all transiently- and stably-produced samples shifted to ~ 32 kDa after treatment (Fig. 4B/C), indicating that the rRBD protein produced by both processes was N-glycosylated [39, 40].

## Discussion

In this work, transient and stable processes for protein production in Expi293F cells were developed by systematic evaluation of both vector elements and culture conditions using rRBD as a model protein. Initial process development was performed at the 24 DWP-scale to quickly identify the vectors that could drive the highest rRBD titer after transient transfection as well as the stable clones with the highest fed-batch titers. Scale-up of both transient and stable processes to shake flask culture verified transient process scalability and demonstrated that the stable rRBD producing clones could be cultured in fed-batch format. Our transient rRBD yields reached up to 100 mg/L by ELISA, which is higher than those reported by many sources [39, 41–44] with the exception of two studies that report similar yields up to 80–100 mg/L[10, 45]. The stable cell lines developed in this work are the first clonal, HEK293-derived rRBD producers described in literature to our knowledge and produced up to 140 mg/L by ELISA. The only other HEK293-based stable rRBD production platforms reported are based on polyclonal pools cultured in batch format [45–47].

Previous work by others to produce rRBD transiently in HEK293 was done using vectors containing a CAG-rRBD [9, 41, 43, 44] or CMV-rRBD [39, 45] cassette transfected into cells grown at 37 °C. A study that compared final protein yields from cultures transfected with a CAG-rRBD vector or one of four CMV-rRBD vectors reported the mean titer driven by the CAG-rRBD vector ranked fourth out of the five constructs screened. Since other vector elements varied when the rRBD promoter was changed, including the sequence of the rRBD protein itself, it was difficult to definitively conclude which promoter is best suited for expression from these data [42]. Our work used the same base vector to explore the effects that the promoter driving rRBD expression had on titer to enable head-to-head comparisons between conditions. We observed that the CAG and CMV promoters drove similar rRBD expression at 37 °C while expression driven by SV40 yielded 0.4-fold less protein. The SV40 promoter has been shown to drive relatively low expression in HEK293 cells [18, 48, 49], but whether the CMV or CAG promoter drives stronger transgene expression appears to be more context dependent. A survey of other studies comparing CMV and CAG driven protein yields at 37 °C shows that CMV-driven expression is stronger for some target protein and HEK293 host combinations [48, 50], while the CAG promoter is stronger for others [19, 20].

In our study, CMV and CAG-driven rRBD expression increased when cells were cultured at 32 °C while SV40-driven expression did not change. These results match previous observations regarding the temperature responsiveness of all three promoters [20, 22], and one study shows the CMV enhancer present in both the CMV and CAG promoters contributes to enhanced expression at 32 °C [22]. However, the effect of reduced culture temperature on recombinant protein titers likely also depends on other factors including the biophysical properties of the target protein [9, 51–53] and characteristics of the host cell line [9, 54]. For instance, production of the full spike protein using the CAG promoter has generally been shown to increase when cells are cultured at 32 °C post-transfection versus 37 °C [9, 54, 55]. However, the corresponding effect on production of rRBD variants seems to depend on the exact amino acid sequence expressed and host platform utilized [9, 42, 51, 56]. Though definitive consensus on which sequences have the most pronounced effects on temperature responsiveness and titers has not yet been reached, it is interesting to note that a rRBD-317–599 variant, which spans a sequence similar to our rRBD-319-599 protein and contains an additional beta sheet compared to the more commonly studied core domain RBD-319-541, was observed to be more easily secreted than other variants [51].

Our transient expression vector system was also used to study the effect of in *cis* co-expression of episomal maintenance factors on rRBD yield. Titers were either similar or lower when WT EBNA1 was co-expressed on CMV-rRBD or CAG-rRBD constructs, regardless of culture temperature. We also observed that in *cis* EBNA1 co-expression did not enhance eGFP expression over a 6-day batch culture (Figure S3A/B). However, a study with induced pluripotent stem cells (iPSCs) showed that the mean expression level and the population of eGFP-positive cells was twice as high after cultures were transfected with vectors co-expressing EBNA1/oriP in *cis* [57]. Additionally, our Expi293F cultures co-expressing EBNA1 showed sustained low-level eGFP expression over multiple weeks, which was not observed for eGFP-only expressing cells (Figure S3C/D). Taken together, these results indicate that the utility of EBV elements to increase protein expression may be cell line and context dependent.

The effect of augmenting transient EBNA1 expression with additional promoter sequences has on transgene expression also appears to be context dependent. We observed that lower mean rRBD titers were achieved when cultures were transfected with vectors containing the sSV40 promoter placed upstream of the wild-type EBNA1 gene versus when transfected with vectors only containing the WT EBNA1 sequence or without EBNA1/oriP. These results suggest that enhancing EBNA1 expression in *cis* provides no meaningful benefit with respect to recombinant protein titers. Interestingly, results from other studies using iPSCs and HEK293-6E suggest that an optimized, transient EBNA1 co-expression cassette may need to be supplied in *trans* to significantly increase transgene expression [30, 57, 58].

While the work described here and other studies have shown that optimized HEK293-based stable processes outperform transient platforms [12–15], stable cell line generation and screening is less straightforward and more time-consuming than scaling up a transient process. Previous literature has shown that relative titer trends generally hold between conditions when transient processes are scaled [59, 60]. However, relative titer rankings of stable cell lines can change throughout clone screening and fed-batch process development due to changes in cell growth or production rates when transitioning between culture scales or upon addition of feeds [61, 62]. This may explain the observed differences in mean titer rankings for the stable clones after proceeding from the batch screen (A5, C3, D3, A3) to the fed-batch production runs (C3, D3, A3, A5). Most notably, in the initial 7-day batch screen, clone A5 produced 1.4-fold more rRBD than clone C3, but in the final-fed batches, C3 produced 1.2-fold more rRBD than A5.

The relative titer differences between clones in this study may have arisen due to differences in growth profiles and stability. Analysis of samples taken on fed-batch day 7, day 14, and on harvest day during pilot batches for all four clones showed that A5 had already reached 50% of its final titer by day 7, while clones C3, D3, and A3 had only produced 20–30% of their respective final titers (Figure S10). A5 exhibited slightly slower growth during exponential phase of the final fed-batches, but otherwise reached peak VCDs similar to those of the other clones (Figure S6A). These observations suggest that the specific productivity of A5 may have dropped over the course of the culture, such that its titer would appear better relative to the other clones in batch but not fed-batch format. All other clones accumulated the majority of the rRBD produced during the second week of culture when cell density was the highest, indicating that their specific productivities are likely more stable over longer culture periods. It is also interesting to note that of the top four clones, C3 grew the slowest while being adapted to suspension culture after single cell cloning (Table S3) but had the highest rRBD copy number (Figure S7) and fed-batch titer (Fig. 3B). This suggests that it may be worth monitoring less vigorous clones that survive the adaptation to suspension for additional passages to determine if they might eventually recover normal growth profiles and display favorable productivity profiles during batch screening. Future work employing site-specific integration (SSI) techniques for HEK293 cell line development instead of solely relying on MAR elements to attenuate positional effects may further increase titer and cell line stability. While SSI methods have been used to develop highly productive CHO cell lines by expressing recombinant proteins at well-characterized genomic loci, only limited work has been done to apply SSI for HEK293 cell line development to date [63, 64].

Although the use of a stable fed-batch platform to produce rRBD in this work was most productive, maximizing absolute protein titers is not the only consideration when selecting a production system. Process duration, scalability, and cost must be considered along with the demand for the target protein [65]. The most productive transient process developed in this work is only 6 days long, which is relatively short compared to the multi-week fed-batch process that is preceded by 2 months of cell line development. However, transient production requires new plasmid DNA and transfection reagents for each run, which amounts to a substantial cost if batches must be run frequently and at large scales. The stable fed-batch process developed in this work could be further optimized by terminating cultures after ~ 2 weeks, as ~ 90% of the rRBD is produced by day 14 (Figure S10). Studies have also shown that yeast-based production platforms can be utilized for antigen production, as engineered rRBD sequences have been produced at titers up to 68 mg/L in 3-day *Pichia pastoris* batches. However, native protein sequences may need to be modified to be produced at appreciable levels in yeast, and post-translational modifications conferred by yeast cells can be less homogeneous than those conferred by mammalian hosts. The suitability of yeast derived proteins for use in medical settings as vaccines and in serology tests must therefore be evaluated against human derived proteins on a case by case basis [66–68].

## Conclusion

This work demonstrates the ability of HEK293-derived cells to produce rRBD-319-599 at levels of ≥ 100 mg/L by ELISA in both transient batch and stable fed-batch production formats. While rapid titer analysis from crude supernatant samples was enabled by a BLI assay during DWP-scale process development, differences in matrix effects from the transient and stable batch supernatants necessitated the use of an ELISA for accurate comparison of titers from the top performing processes of each platform type. The highest transient rRBD titer, 100 mg/L by ELISA, was obtained when cells were transfected with vectors expressing rRBD under the CMV promoter followed by culture at 32 °C. Co-expression of EBNA1 and oriP in *cis* did not significantly increase transgene titers over the 6-day transient batch period. Clonal cell lines expressing a stably integrated rRBD expression cassette produced rRBD up to 140 mg/L by ELISA in 20–24 day fed-batches at 37 °C. While both transient and stable processes are suitable for rRBD production in Expi293F, an optimized stable process would be the most productive and economically viable option for long-term protein production at large scales.

## Electronic supplementary material

Below is the link to the electronic supplementary material.

Supplementary Material 1

Supplementary Material 2

Supplementary Material 3

## Data Availability

All data generated or analyzed during this study are included in this published article and its supplementary information files or available upon reasonable request. Additional File 1, Supplemental Information, contains Tables S1–S3 and Figures S1–S10 referenced throughout the manuscript. Additional File 2, Sequence Information for rRBD Plasmids, contains Table S4, which describes how to access full rRBD plasmid sequences via GenBank. Additional File 3, Original Protein Gel Images, contains Figures S11–S13, which are the uncropped, original protein gel images shown in Fig. 4.
