# Supplementary material for: Comparison of vector elements and process conditions in transient and stable suspension HEK293 platforms using SARS-CoV-2 receptor binding domain as a model protein
Source: BMC Biotechnol. 2023 Mar 7;23:7. doi: 10.1186/s12896-023-00777-7 (PMC9990576; doi:10.1186/s12896-023-00777-7)
Supplement: Supplementary file 1 — Supplementary Material 1 [file 12896_2023_777_MOESM1_ESM.pdf]

Additional File 1: Supplemental Information

Comparison of vector elements and process conditions for transient and stable production of SARS-CoV-2 receptor binding domain in suspension HEK293 cells

Erica A. Green[1], Nathaniel K. Hamaker[1], and Kelvin H. Lee[1]

[1]Department of Chemical and Biomolecular Engineering, University of Delaware, 590 Avenue 1743, Newark, Delaware, 19713, USA

Corresponding author: Lee, Kelvin H. (KHL@udel.edu)

This work was supported in part by the financial assistance awards 70NANB17H002 and 70NANB21H085 from U.S. Department of Commerce, National Institute of Standards and Technology. EAG was funded in part by NIH NIGMS T32GM133395 and NKH was funded in part by NIH NIGMS T32GM008550 from the National Institute of General Medical Sciences.

Table S1: Primer/probe assay sequences used for recombinant receptor binding domain (rRBD) copy number analysis

Name	Forward primer (5'-3')	Reverse primer (5'-3')	Probe (5'-3')
rRBD set 1	GCAAGATCGCTGACT ACAACTA	TTCCGCCGACTTTGCT ATC	/56-FAM/ATGTGTCAT /ZEN/CGCCTGGAACA G CAA/3IABkFQ/
rRBD set 2	AGCGTTTACGCCTGG AATAG	ACAGGTCGTTCAGCT TTGTAG	/56-FAM/AATCGGCC A/Zen/CGCAGTTAGA GATCC/3IABkFQ/
GAPDH	GGGAAGCTCAAGGG A GATAAA	CAGAATATGTGAGCA GCCCTA	/5HEX/TAAGAGGG C/Zen/GAATGCAGCA TCTCC/3IABkFQ/
RPP30	GCATATCAGGGTACA GCATAGG	CTTCCCTCACGGCAT ATACTTC	/5HEX/TCTGCTCGT /ZEN/TGTTAGTCACCA GCT/3IABkFQ/

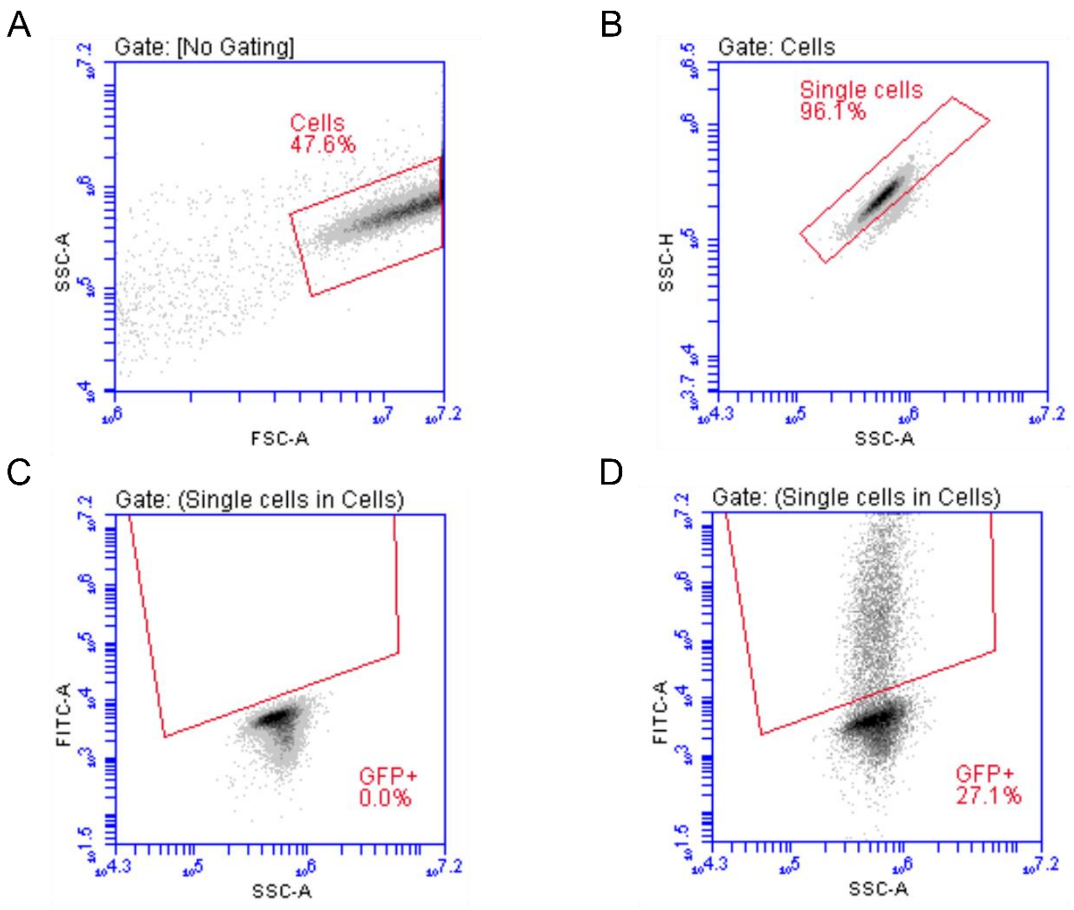

Figure S1: Flow cytometry gating strategy for tracking enhanced green fluorescent protein positive (eGFP+) cell populations

(A) Cells were isolated from debris by applying a gate on a plot of side scatter-area (SSC-A) vs forward scatter-area (FSC-A). **(B)** Single cells were isolated by applying a gate on a plot of SSC-height (SSC-H) vs SSC-A. **(C)** Untransfected host cells were used to draw a final gate on a plot of fluorescence-area (FITC-A) versus SSC-A to isolate eGFP+ cells. **(D)** eGFP+ cells on a plot of FITC-A vs SSC-A one day after transfection with pCXLE-EGFP and culture at 32°C.

Table S2: Absolute bio-layer interferometry (BLI) titers used to normalize transient and stable deep well plate (DWP) batch titers

Batch	Normalization condition	Absolute titer value (mg/L) ± SEM	Dilution factor
Transient DWP-1 (Figure 1B)	rRBD-CAG, 37°C	83.9 ± 8.7	2
Transient DWP-2 (Figure 1C)	rRBD-CAG, 37°C	18.1 ± 0.2	5
Transient DWP-3 (Figure 1D)	rRBD-CAG, 32°C	219.6 ± 19.0	2
Stable clone DWP (Figure 2)	Selected pool, 37°C	41.4 ± 1.7	2

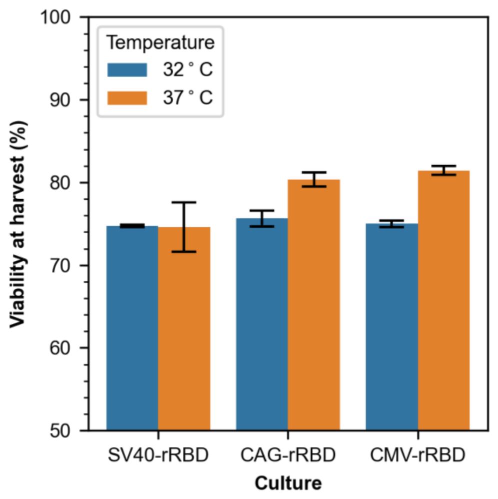

Figure S2: Viabilities of transient DWP cultures at harvest

Harvest day cell viabilities for the first DWP study investigating the effect of temperature and rRBD promoter on titer. Cultures were harvested when viability was <80%, which occurred on day 3 and day 6 post transfection for cultures grown at 37°C and 32°C, respectively. Error bars show SEM for biological duplicates.

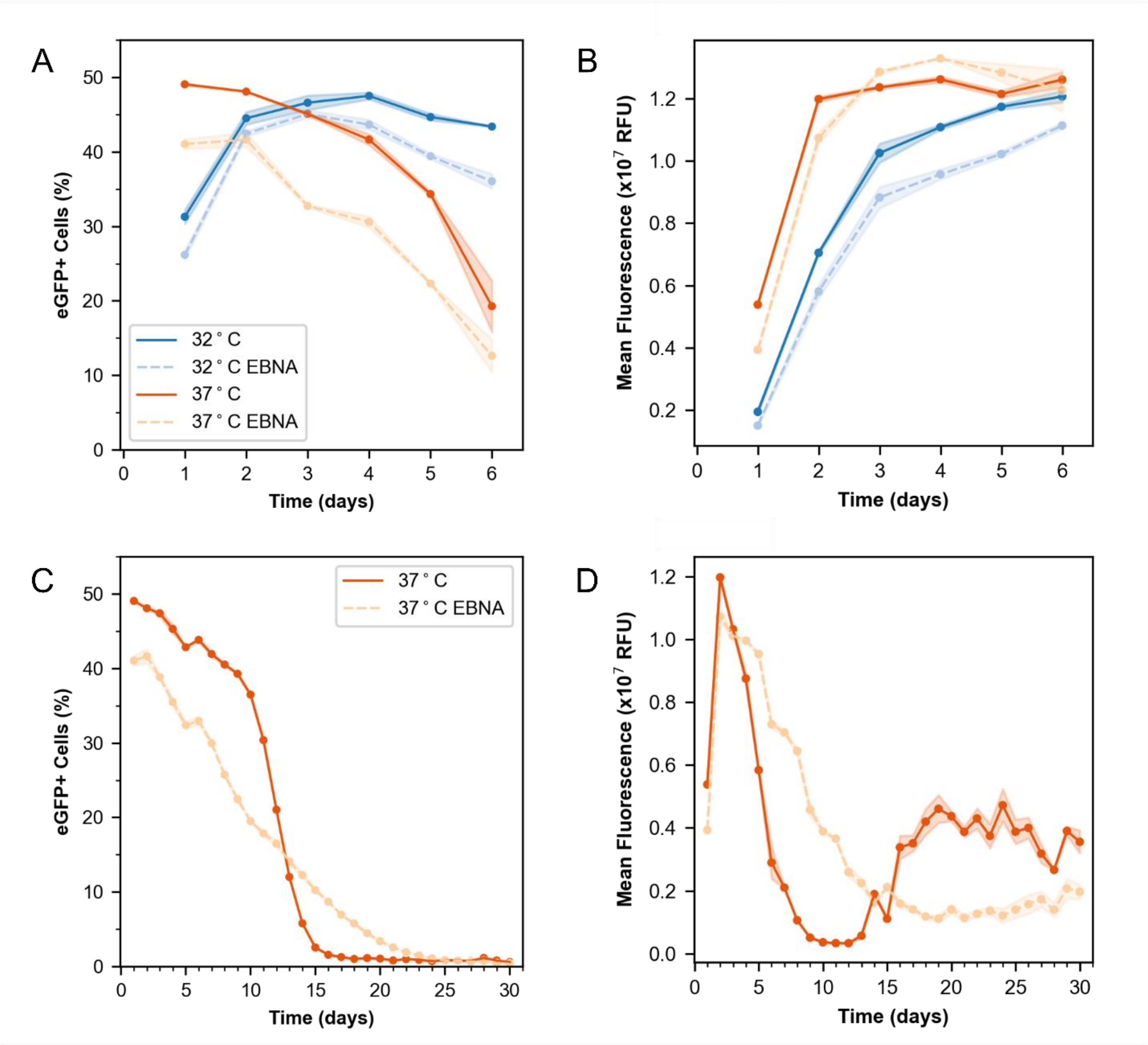

Figure S3: eGFP expression profiles over a transient batch and long-term culture with and without EBNA1 co-expression.

(S3A/B): Trends for **(A)** the percentage of eGFP positive (eGFP+) cells and **(B)** the mean fluorescence intensity (MFI) of eGFP+ cells over a 6-day batch. Cultures were grown at 32°C (blue line) or 37°C (orange line) and vectors either did (light dashed line) or did not (dark solid line) contain the Epstein-Barr virus (EBV) nuclear antigen 1 (EBNA1) gene. **(S3C/D):** Trends for **(C)** the percentage of eGFP+ cells and **(D)** the MFI of eGFP+ cells over 30 days in culture. Cultures were grown at 37°C, and vectors either did (light orange line) or did not (dark orange line) contain the EBNA1 gene. Error banding shows SEM for biological triplicates.

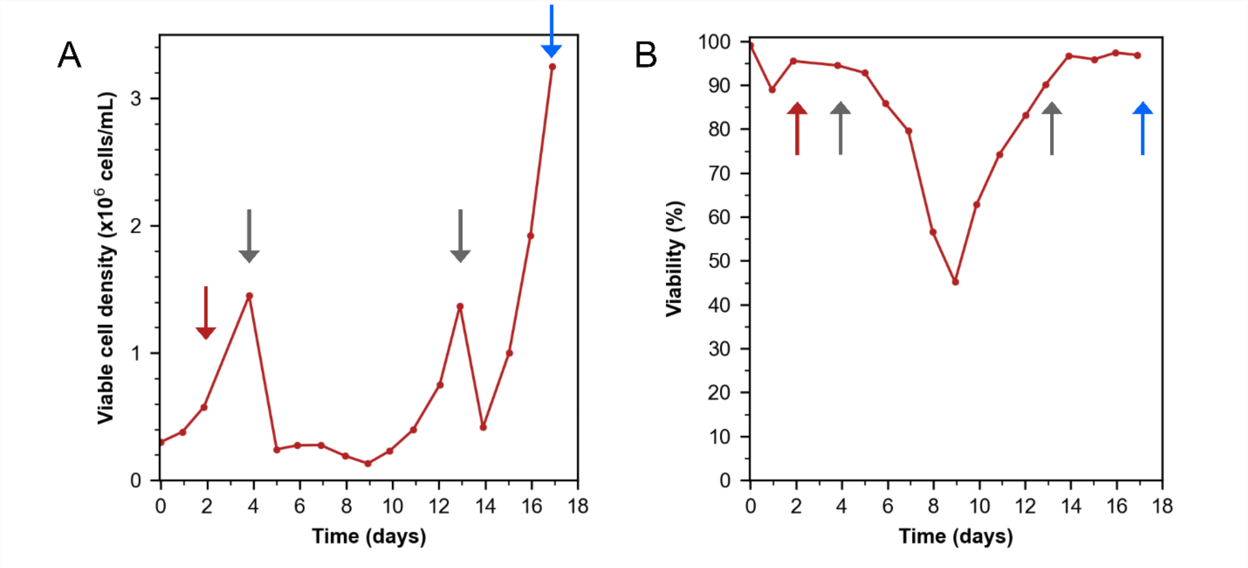

Figure S4: Viable cell density (VCD) and viability curves for selection of the stable rRBD expressing pool

(A) VCD and **(B)** viability trends for Expi293F cells during selection of the rRBD stable pool. Geneticin was added day 2 post-transfection (red arrow). Cells were passaged if VCD > 1 x 10^6 cells/mL (grey arrows) until VCD and viability recovered to match those of untransfected host cells (blue arrow).

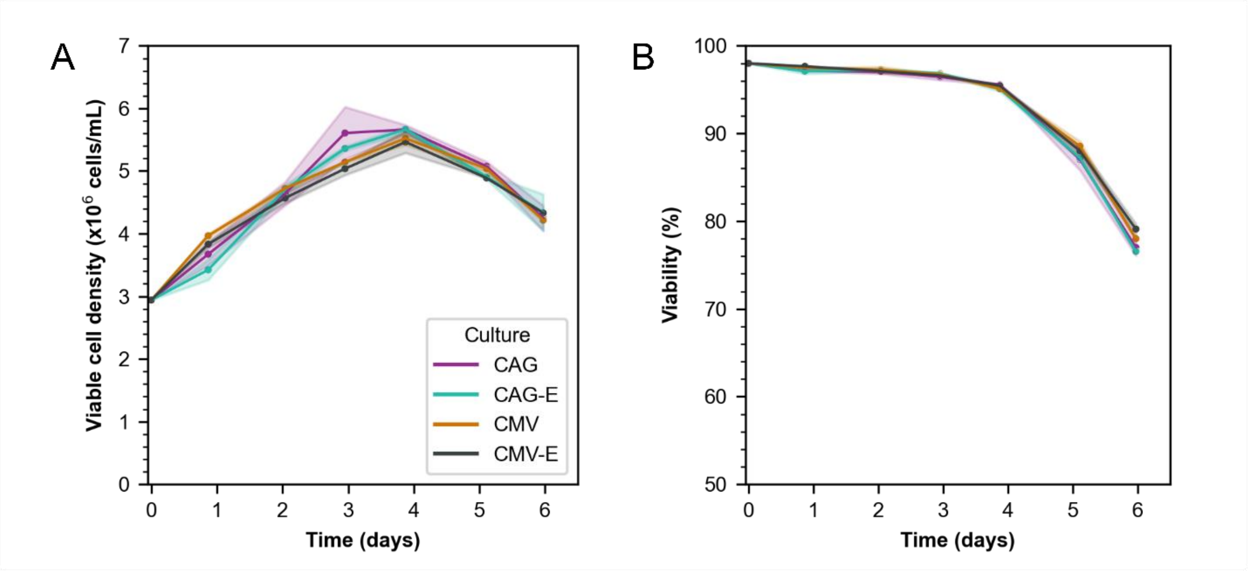

Figure S5: VCD and viability curves for transient rRBD shake flask batches

(A) VCD and **(B)** viability trends for the 125 mL transient shake flask batches. The legend denotes the promoter used for rRBD expression and whether the vector contained the oriP/wild-type EBNA1 sequences (-E). Error banding shows SEM for biological duplicates.

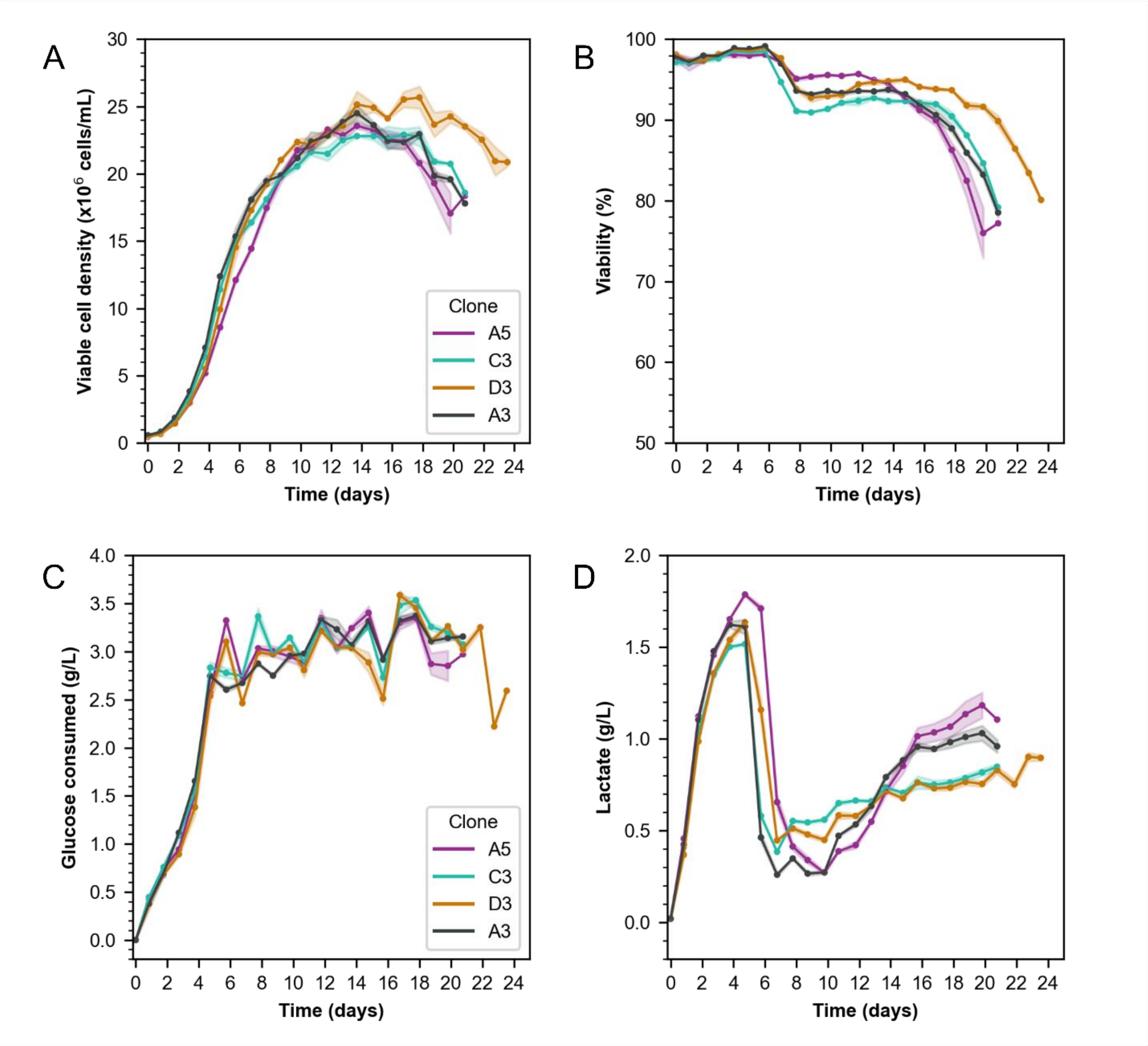

Figure S6: Cell growth and metabolite profiles for the top clone fed-batches in shake flasks

Trends for **(A)** VCD, **(B)** viability, **(C)** glucose consumption, and **(D)** lactate levels during the fed-batches. Glucose consumption was calculated by subtracting each day's glucose reading from the daily re-feed set point (~7g/L) or previous day's reading if not fed. Error banding shows SEM for biological triplicates.

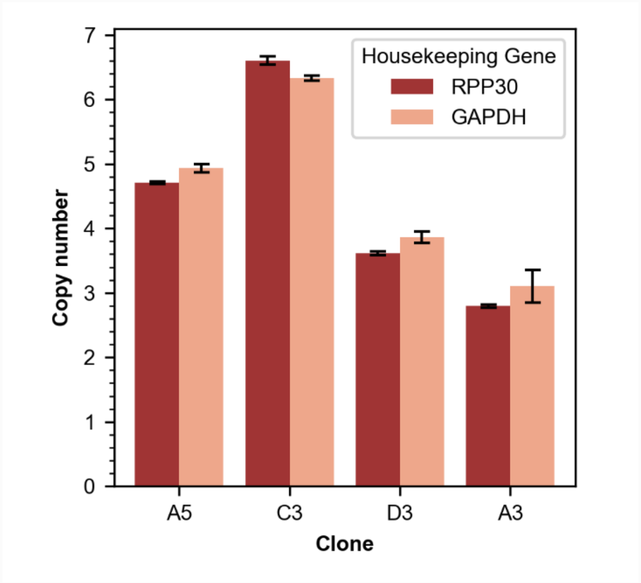

Figure S7: Copy number analysis of the top four clones by droplet digital polymerase chain reaction (ddPCR)

DNA copy numbers of the top rRBD clonal cell lines as measured by ddPCR against two different housekeeping genes, *RPP30* and *GAPDH*. Error bars show SEM for technical triplicates.

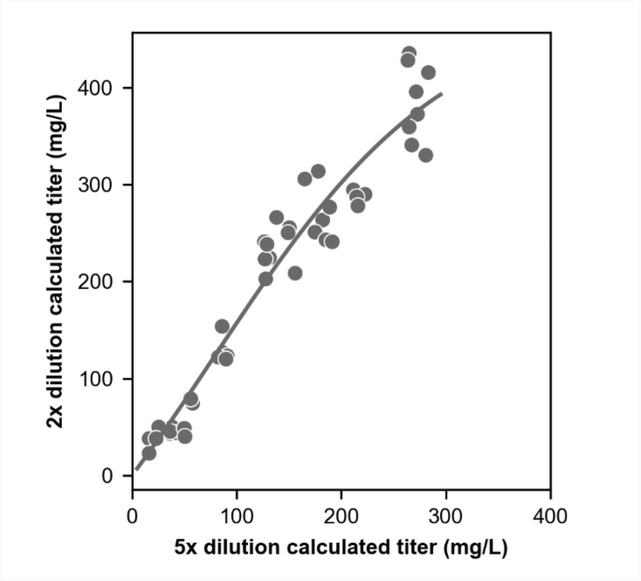

Figure S8: 4-parameter logistic (4PL) relationship for Anti-Penta-HIS Octet BLI titer measurements by dilution factor

Select transiently produced rRBD samples from all DWP and flask-scale batches were measured at 2- and 5-fold dilutions to explore the extent to which matrix effects at different dilutions contribute to differences in BLI titer readings. Titer measurements taken on 2- and 5-fold diluted samples and subsequently corrected for dilution factor are shown on the y and x axes, respectively. Each point shows a single technical replicate measurement.

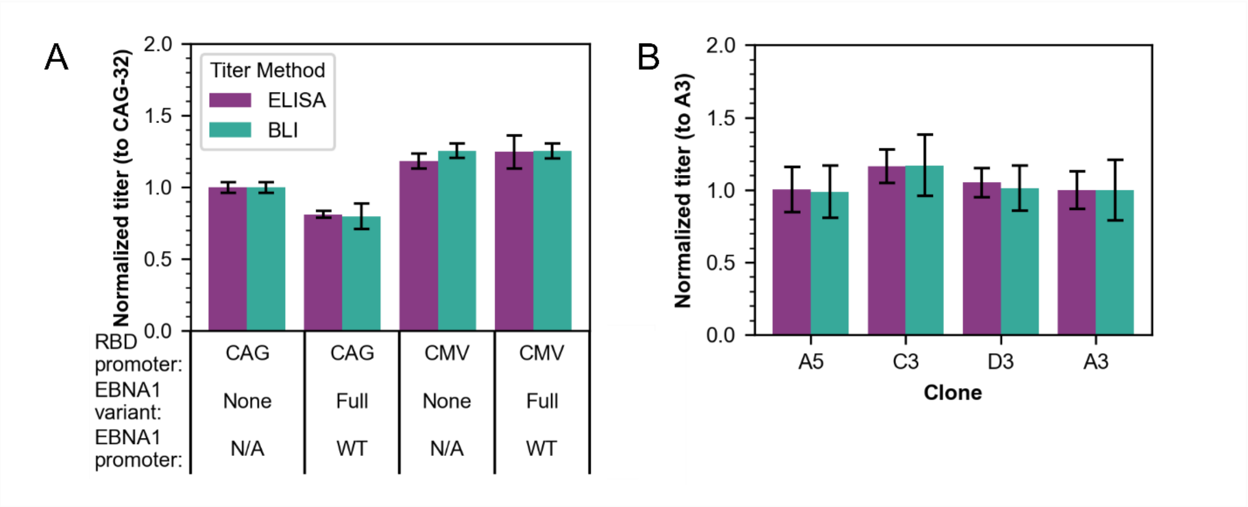

Figure S9: Normalized titers for 125 mL flask-scale batches measured by orthogonal analytical methods

Normalized titers **(S9A-** transient**, S9B-** stable**)** measured on harvested supernatant enzyme-linked immunosorbent assay (ELISA) and BLI for the flask scale batches.

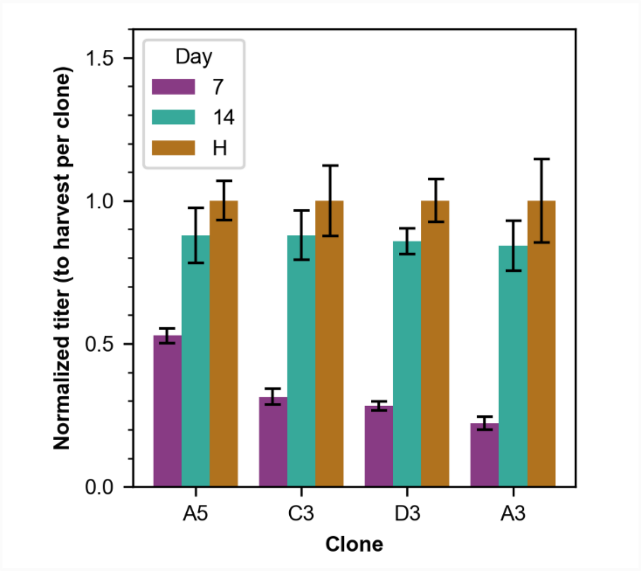

Figure S10: Weekly tracking of fed-batch titers measured by BLI during pilot batches

Titers of fed-batch samples taken weekly during process development were measured to track how protein accumulates over the culture duration. All titers are normalized to the harvest day (H) titer for each individual clone (day 20 for A5/C3, and day 23 for A3/D3). Error bars show SEM on technical duplicates.

Table S3: VCD, viability, and batch/fed-batch titer rankings for clones that exhibited outgrowth from single-cell cloning after two passages at 24 DWP scale

Clone	VCD ($x10^6$ cells/mL)	Viability (%)	Batch screen titer rank	Fed-batch titer rank
A5	2.9	96.7	1	4
C3	1.25	93.6	2	1
D3	2.4	98.3	3	2
A3	4.73	98.8	4	3
D6	2.49	98.4	5	--
C1	4.13	99.5	6	--
B2	2.91	98.4	7	--
A2	2.87	98.5	8	--
D2	3.18	98.9	9	--
C6	3.69	99.3	10	--
D1	2.49	96.8	11	--
B6	2.89	98.2	12	--
D4	2.9	99.3	13	--
A4	0.16	90.7	--	--
A6	0.608	90.5	--	--
B3	0.099	85.7	--	--
C2	0.304	86	--	--
D5	0.118	86.2	--	--

"--" indicates that the clone was not cultured at the indicated screen stage
