# Supplementary material for: Comparison of vector elements and process conditions in transient and stable suspension HEK293 platforms using SARS-CoV-2 receptor binding domain as a model protein
Source: BMC Biotechnol. 2023 Mar 7;23:7. doi: 10.1186/s12896-023-00777-7 (PMC9990576; doi:10.1186/s12896-023-00777-7)
Supplement: Supplementary file 2 — Supplementary Material 2 [file 12896_2023_777_MOESM2_ESM.pdf]

Additional File 2: Sequence Information for rRBD Plasmids

Comparison of vector elements and process conditions for transient and stable production of SARS-CoV-2 receptor binding domain in suspension HEK293 cells

Erica A. Green[1], Nathaniel K. Hamaker[1], and Kelvin H. Lee[1]

[1]Department of Chemical and Biomolecular Engineering, University of Delaware, 590 Avenue 1743, Newark, Delaware, 19713, USA

Corresponding author: Lee, Kelvin H. (KHL@udel.edu)

This work was supported in part by the financial assistance awards 70NANB17H002 and 70NANB21H085 from U.S. Department of Commerce, National Institute of Standards and Technology. EAG was funded in part by NIH NIGMS T32GM133395 and NKH was funded in part by NIH NIGMS T32GM008550 from the National Institute of General Medical Sciences.

Table S4: Accession numbers for rRBD plasmid sequences available via GenBank, the NIH genetic sequence database

Plasmid name	GenBank Accession Number[1]
pSV40-rRBD	OP697985
pCAG-rRBD	OP697986
pCAG-rRBD-wtEBNA1	OP697987
pCAG-rRBD-xrepEBNA1	OP697988
pCAG-rRBD-svEBNA1	OP697989
pCMV-rRBD	OP697990
pCMV-rRBD-wtEBNA1	OP697991
pCMV-rRBD-xrepEBNA1	OP697992
pCMV-rRBD-svEBNA1	OP697993
pNeo-CAG-rRBD	OP697994

[1]GenBank can be accessed at https://www.ncbi.nlm.nih.gov/genbank/
