# Supplementary material for: Comparison of vector elements and process conditions in transient and stable suspension HEK293 platforms using SARS-CoV-2 receptor binding domain as a model protein
Source: BMC Biotechnol. 2023 Mar 7;23:7. doi: 10.1186/s12896-023-00777-7 (PMC9990576; doi:10.1186/s12896-023-00777-7)
Supplement: Supplementary file 3 — Supplementary Material 3 [file 12896_2023_777_MOESM3_ESM.pdf]

Additional File 3: Original Protein Gel Images

Comparison of vector elements and process conditions for transient and stable production of SARS-CoV-2 receptor binding domain in suspension HEK293 cells

Erica A. Green[1], Nathaniel K. Hamaker[1], and Kelvin H. Lee[1]

[1]Department of Chemical and Biomolecular Engineering, University of Delaware, 590 Avenue 1743, Newark, Delaware, 19713, USA

Corresponding author: Lee, Kelvin H. (KHL@udel.edu)

This work was supported in part by the financial assistance awards 70NANB17H002 and 70NANB21H085 from U.S. Department of Commerce, National Institute of Standards and Technology. EAG was funded in part by NIH NIGMS T32GM133395 and NKH was funded in part by NIH NIGMS T32GM008550 from the National Institute of General Medical Sciences.

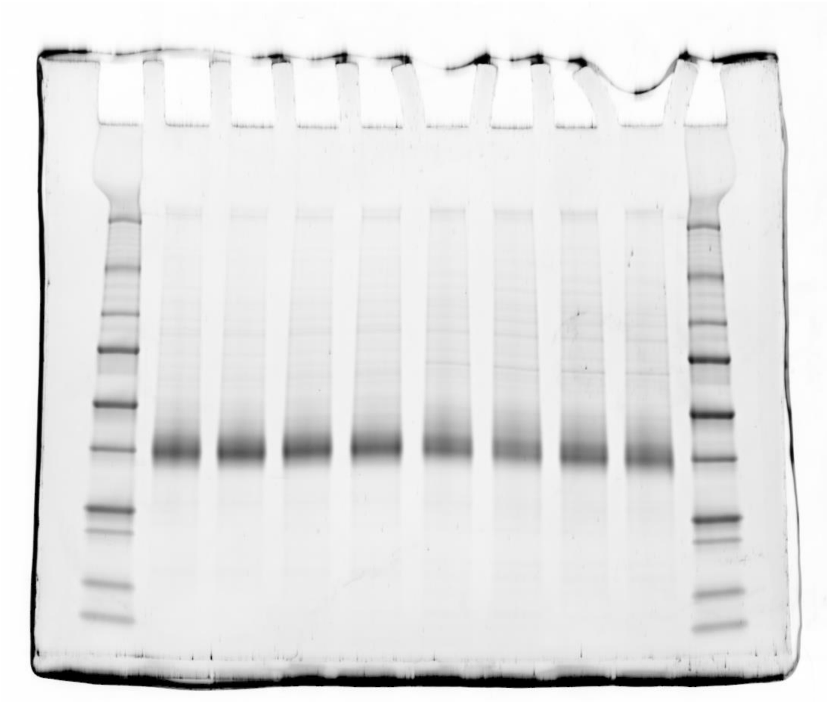

Figure S11: Uncropped and unlabeled protein gel image from main text Figure 4A

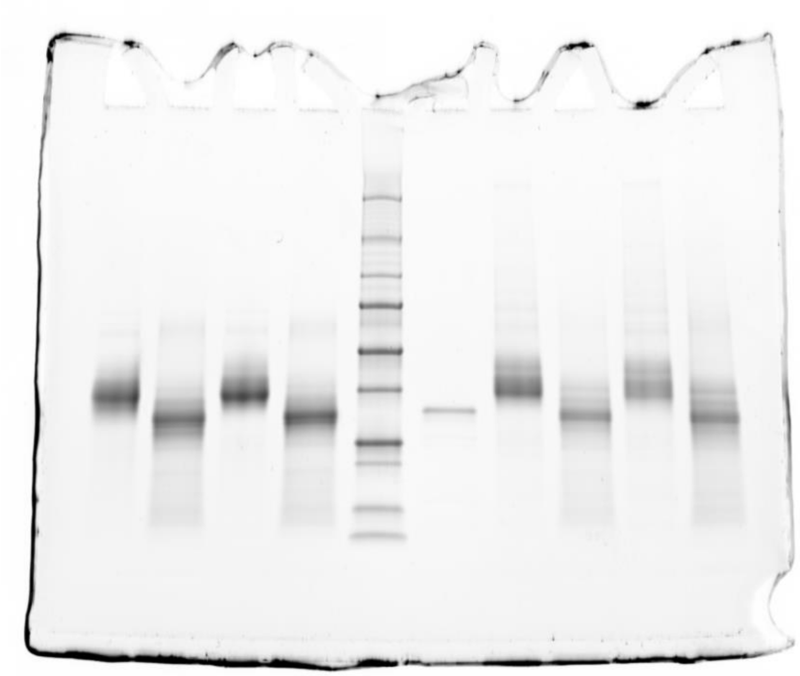

Figure S12: Uncropped and unlabeled protein gel image from main text Figure 4B

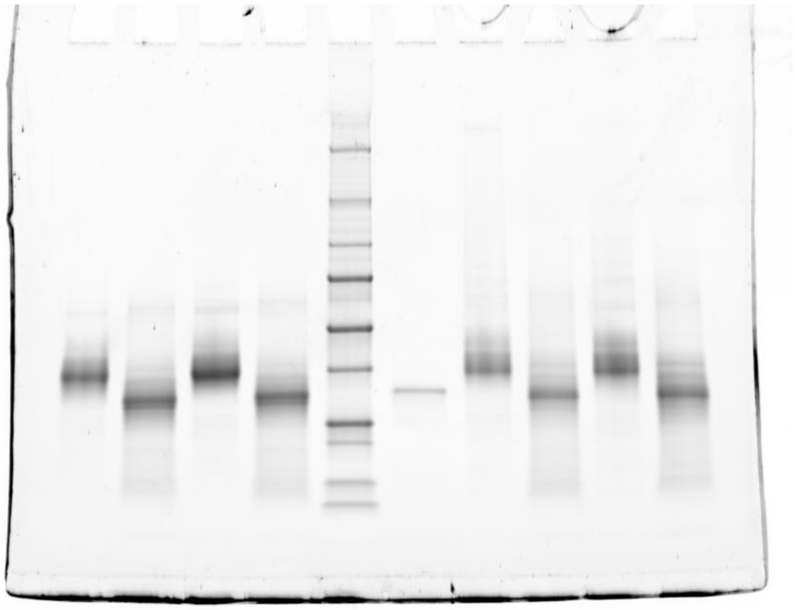

Figure S13: Uncropped and unlabeled protein gel image from main text Figure 4C
